# COSORE: A community database for continuous soil respiration and other soil‐atmosphere greenhouse gas flux data

**DOI:** 10.1111/gcb.15353

**Published:** 2020-10-07

**Authors:** Ben Bond‐Lamberty, Danielle S. Christianson, Avni Malhotra, Stephanie C. Pennington, Debjani Sihi, Amir AghaKouchak, Hassan Anjileli, M. Altaf Arain, Juan J. Armesto, Samaneh Ashraf, Mioko Ataka, Dennis Baldocchi, Thomas Andrew Black, Nina Buchmann, Mariah S. Carbone, Shih‐Chieh Chang, Patrick Crill, Peter S. Curtis, Eric A. Davidson, Ankur R. Desai, John E. Drake, Tarek S. El‐Madany, Michael Gavazzi, Carolyn‐Monika Görres, Christopher M. Gough, Michael Goulden, Jillian Gregg, Omar Gutiérrez del Arroyo, Jin‐Sheng He, Takashi Hirano, Anya Hopple, Holly Hughes, Järvi Järveoja, Rachhpal Jassal, Jinshi Jian, Haiming Kan, Jason Kaye, Yuji Kominami, Naishen Liang, David Lipson, Catriona A. Macdonald, Kadmiel Maseyk, Kayla Mathes, Marguerite Mauritz, Melanie A. Mayes, Steve McNulty, Guofang Miao, Mirco Migliavacca, Scott Miller, Chelcy F. Miniat, Jennifer G. Nietz, Mats B. Nilsson, Asko Noormets, Hamidreza Norouzi, Christine S. O’Connell, Bruce Osborne, Cecilio Oyonarte, Zhuo Pang, Matthias Peichl, Elise Pendall, Jorge F. Perez‐Quezada, Claire L. Phillips, Richard P. Phillips, James W. Raich, Alexandre A. Renchon, Nadine K. Ruehr, Enrique P. Sánchez‐Cañete, Matthew Saunders, Kathleen E. Savage, Marion Schrumpf, Russell L. Scott, Ulli Seibt, Whendee L. Silver, Wu Sun, Daphne Szutu, Kentaro Takagi, Masahiro Takagi, Munemasa Teramoto, Mark G. Tjoelker, Susan Trumbore, Masahito Ueyama, Rodrigo Vargas, Ruth K. Varner, Joseph Verfaillie, Christoph Vogel, Jinsong Wang, Greg Winston, Tana E. Wood, Juying Wu, Thomas Wutzler, Jiye Zeng, Tianshan Zha, Quan Zhang, Junliang Zou

**Affiliations:** ^1^ Pacific Northwest National Laboratory Joint Global Change Research Institute at the University of Maryland–College Park College Park MD USA; ^2^ Lawrence Berkeley National Laboratory Berkeley CA USA; ^3^ Department of Earth System Science Stanford University Stanford CA USA; ^4^ Climate Change Science Institute and Environmental Sciences Division Oak Ridge National Laboratory Oak Ridge TN USA; ^5^ Department of Civil and Environmental Engineering University of California Irvine Irvine CA USA; ^6^ School of Geography and Earth Sciences McMaster University Hamilton Ontario Canada; ^7^ Departamento de Ecología Pontificia Universidad Católica de Chile Santiago Chile; ^8^ Instituto de Ecología y Biodiversidad Santiago Chile; ^9^ Department of Building, Civil and Environmental Engineering Concordia University Montreal QC Canada; ^10^ Research Institute for Sustainable Humanosphere Kyoto University Uji City Kyoto Japan; ^11^ Department of Environmental Science, Policy, and Management University of California Berkeley CA USA; ^12^ Faculty of Land and Food Systems University of British Columbia Vancouver BC Canada; ^13^ Department of Environmental Systems Science Institute of Agricultural Sciences ETH Zurich Zurich Switzerland; ^14^ Center for Ecosystem Science and Society Northern Arizona University Flagstaff AZ USA; ^15^ Department of Natural Resources and Environmental Studies Center for Interdisciplinary Research on Ecology and Sustainability National Dong Hwa University Hualien Taiwan; ^16^ Department of Geological Sciences and Bolin Centre for Climate Research Stockholm University Stockholm Sweden; ^17^ Department of Evolution, Ecology and Organismal Biology Ohio State University Columbus OH USA; ^18^ Appalachian Laboratory University of Maryland Center for Environmental Science Frostburg MD USA; ^19^ Department of Atmospheric and Oceanic Sciences University of Wisconsin‐Madison Madison WI USA; ^20^ Sustainable Resources Management SUNY‐ESF Syracuse NY USA; ^21^ Max Planck Institute for Biogeochemistry Jena Germany; ^22^ Eastern Forest Environmental Threat Assessment Center USDA Forest Service Research Triangle Park NC USA; ^23^ Plants and Ecosystems Department of Biology University of Antwerp Wilrijk Belgium; ^24^ Department of Biology Virginia Commonwealth University Richmond VA USA; ^25^ University of California at Irvine Irvine CA USA; ^26^ Sustainability Double Degree Program Oregon State University Corvallis OR USA; ^27^ Institute of Ecology College of Urban and Environmental Sciences Peking University Beijing China; ^28^ Research Faculty of Agriculture Hokkaido University Sapporo Japan; ^29^ Pacific Northwest National Laboratory Richland WA USA; ^30^ Smithsonian Environmental Research Center Edgewater MD USA; ^31^ School of Forest Resources University of Maine Orono ME USA; ^32^ Department of Forest Ecology and Management Swedish University of Agricultural Sciences Umeå Sweden; ^33^ Beijing Research & Development Centre for Grass and Environment Beijing Academy of Agriculture and Forestry Sciences Beijing China; ^34^ The Pennsylvania State University University Park PA USA; ^35^ Forestry and Forest Products Research Institute Tsukuba‐city Japan; ^36^ Center for Global Environmental Research National Institute for Environmental Studies Tsukuba Japan; ^37^ Biology Department San Diego State University San Diego CA USA; ^38^ Hawkesbury Institute for the Environment Western Sydney University Penrith NSW Australia; ^39^ School of Environment, Earth and Ecosystem Sciences The Open University Milton Keynes UK; ^40^ Integrated Life Sciences Virginia Commonwealth University Richmond VA USA; ^41^ Biological Sciences University of Texas at El Paso El Paso TX USA; ^42^ School of Geographical Sciences Fujian Normal University Fuzhou P.R. China; ^43^ University at Albany State University of New York New York NY USA; ^44^ USDA Forest Service Southern Research Station Coweeta Hydrologic Lab Otto NC USA; ^45^ Department of Ecology and Conservation Biology Texas A&M University College Station TX USA; ^46^ New York City College of Technology and the Graduate Center The City University of New York New York NY USA; ^47^ Department of Environmental Studies Macalester College St Paul MN USA; ^48^ UCD School of Biology and Environmental Science and UCD Earth Institute University College Dublin Dublin Ireland; ^49^ Department of Agronomy University of Almería Almería Spain; ^50^ Department of Environmental Science and Renewable Natural Resources University of Chile Santiago Chile; ^51^ Institute of Ecology and Biodiversity Santiago Chile; ^52^ USDA Agricultural Research Service Forage Seed and Cereal Research Unit Corvallis OR USA; ^53^ Department of Biology Indiana University Bloomington IN USA; ^54^ Department of Ecology, Evolution & Organismal Biology Iowa State University Ames IA USA; ^55^ Institute of Meteorology and Climate Research–Atmospheric Environmental Research KIT‐Campus Alpin Karlsruhe Institute of Technology Garmisch‐Partenkirchen Germany; ^56^ Department of Applied Physics University of Granada Granada Spain; ^57^ School of Natural Sciences Botany Department Trinity College Dublin Dublin Ireland; ^58^ Woods Hole Research Center Falmouth MA USA; ^59^ Southwest Watershed Research Center USDA‐ARS Tucson AZ USA; ^60^ Department of Atmospheric and Oceanic Sciences University of California Los Angeles Los Angeles CA USA; ^61^ Department of Global Ecology Carnegie Institution for Science Stanford CA USA; ^62^ Field Science Center for Northern Biosphere Hokkaido University Horonobe Japan; ^63^ Faculty of Agriculture University of Miyazaki Miyazaki Japan; ^64^ Graduate School of Life and Environmental Sciences Osaka Prefecture University Sakai Japan; ^65^ Department of Plant and Soil Sciences University of Delaware Newark DE USA; ^66^ Department of Earth Sciences and Institute for the Study of Earth, Oceans and Space University of New Hampshire Durham NH USA; ^67^ University of Michigan Biological Station Pellston MI USA; ^68^ Key Laboratory of Ecosystem Network Observation and Modeling Institute of Geographic Sciences and Natural Resources Research Chinese Academy of Sciences Beijing China; ^69^ Department of Science, Engineering and Mathematics Cypress College Cypress CA USA; ^70^ USDA Forest Service International Institute of Tropical Forestry Río Piedras Puerto Rico; ^71^ School of Soil and Water Conservation Beijing Forestry University Beijing P.R. China; ^72^ State Key Laboratory of Water Resources and Hydropower Engineering Science Wuhan University Wuhan P.R. China; ^73^ Present address: Department of Environmental Sciences Emory University Atlanta GA USA; ^74^ Present address: Arid Land Research Center Tottori University Tottori 680–0001 Japan

**Keywords:** carbon dioxide, greenhouse gases, methane, open data, open science, soil respiration

## Abstract

Globally, soils store two to three times as much carbon as currently resides in the atmosphere, and it is critical to understand how soil greenhouse gas (GHG) emissions and uptake will respond to ongoing climate change. In particular, the soil‐to‐atmosphere CO_2_ flux, commonly though imprecisely termed soil respiration (*R*_S_), is one of the largest carbon fluxes in the Earth system. An increasing number of high‐frequency *R*_S_ measurements (typically, from an automated system with hourly sampling) have been made over the last two decades; an increasing number of methane measurements are being made with such systems as well. Such high frequency data are an invaluable resource for understanding GHG fluxes, but lack a central database or repository. Here we describe the lightweight, open‐source COSORE (COntinuous SOil REspiration) database and software, that focuses on automated, continuous and long‐term GHG flux datasets, and is intended to serve as a community resource for earth sciences, climate change syntheses and model evaluation. Contributed datasets are mapped to a single, consistent standard, with metadata on contributors, geographic location, measurement conditions and ancillary data. The design emphasizes the importance of reproducibility, scientific transparency and open access to data. While being oriented towards continuously measured *R*_S_, the database design accommodates other soil‐atmosphere measurements (e.g. ecosystem respiration, chamber‐measured net ecosystem exchange, methane fluxes) as well as experimental treatments (heterotrophic only, etc.). We give brief examples of the types of analyses possible using this new community resource and describe its accompanying R software package.

## Graphical abstract

Here we describe the lightweight, open source COSORE (COntinuous SOil REspiration) database and software. COSORE focuses on automated, continuous and long‐term greenhouse gas flux datasets, and is intended to serve as a community resource for earth sciences, climate change syntheses and model evaluation.

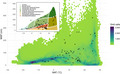

## 1 INTRODUCTION

Fluxes of greenhouse gases (GHGs) between soils and the atmosphere constitute a significant component of global carbon and biogeochemical cycling (Friedlingstein et al., 2019), with the two most commonly measured being those of carbon dioxide (usually referred to as soil respiration, *R*_S_) and methane. Soil respiration constitutes one of the largest carbon fluxes in the entire Earth system (Bond‐Lamberty, 2018; Raich & Potter, 1995; Xu & Shang, 2016) and is useful, but underutilized for constraining and understanding other components of the carbon cycle (Barba et al., 2018; Davidson et al., 2002; Phillips et al., 2017; Wang et al., 2017). Atmospheric methane causes higher 100 year radiative forcing on a mass basis relative to carbon dioxide (Neubauer & Megonigal, 2015) and its production exhibits high temporal and spatial variability often associated with redox conditions (Tang et al., 2016) and climate. This contributes substantial uncertainty to global methane budgets (Friedlingstein et al., 2019; Kirschke et al., 2013; Saunois et al., 2016; Tian et al., 2015). Other GHG fluxes are also measured, albeit less frequently, e.g. nitrous oxide (Gruber & Galloway, 2008), and researchers are beginning to measure multiple gases concurrently as well (Courtois et al., 2019).

These GHG fluxes are measured using a number of techniques (Pumpanen et al., 2004), most commonly infrared gas analyzers (IRGAs; Detto et al., 2011; DuBois et al., 1952) connected to chambers that sit on collars shallowly embedded into the soil surface (Nay et al., 1994; Xu et al., 2006). Continuous measurements of *R*_S_ can also be made using in situ solid‐state sensors (Hirano et al., 2003; Jassal et al., 2005; Tang et al., 2003) and forced diffusion technology (Lavoie et al., 2012, 2015). In the last 30 years, continuously operating automated systems multiplexing multiple chambers to a single IRGA have been developed (Goulden & Crill, 1997; Irvine & Law, 2002; Rayment & Jarvis, 1997). Laser‐based and spectroscopic methods for non‐CO_2_ gases are also increasingly used in field research (Brannon et al., 2016; Savage et al., 2014). These high frequency data, particularly when paired with complementary observations, open up new possible research applications, including understanding rapid plant‐soil ecohydrological links (Volkmann et al., 2016), the coupling of phenology and respiration (Järveoja et al., 2018; Migliavacca et al., 2015; Raich, 2017), the contribution of root respiration (Högberg et al., 2001; Subke et al., 2006), validation of eddy covariance measurements in complex ecosystems (Miao et al., 2017), responses of soil GHG emissions to extreme climate events (Petrakis et al., 2017) and rising atmospheric carbon dioxide concentrations (Drake et al., 2018) and novel inversion techniques (Latimer & Risk, 2016).

The resulting GHG flux datasets, however, remain widely dispersed and frequently unavailable. There is no centralized database for chamber fluxes akin to FLUXNET (Baldocchi et al., 2001), although annual (Bond‐Lamberty & Thomson, 2010) and some daily to seasonal (Jian, Steele, Day, et al., 2018; Jian, Steele, Thomas, et al., 2018) *R*_S_ flux databases do exist. This is troubling, both because of the lost or unavailable research opportunities for synthetic work with respect to temporally high‐resolution GHG fluxes, but also because of the inevitable loss of data (Wolkovich et al., 2012). Fortunately, the tools and knowledge to support a ground‐up community GHG flux database are now available (Lowndes et al., 2017). Here we describe an open database, COSORE (originally derived from ‘COntinuous SOil Respiration’), that focuses on continuous and long‐term soil‐atmosphere GHG flux datasets and is intended to serve as a community resource for future synthesis and model evaluation.

## 2 METHODS

COSORE is designed to be a relatively lightweight database: as simple as possible, but not simpler. It is targeted at continuous—i.e. measured by automated systems—soil respiration flux data, but the database design accommodates manual point (survey‐style) *R*_S_ fluxes, methane fluxes and chamber measurements of net ecosystem exchange as well, paralleling the recent Soil Incubation Database database (Schädel et al., 2020). Its development started in April 2019, and as of this writing (2020‐09‐04) the COSORE version number is 0.8.0.

### 2.1 Database and dataset structure

The database is structured as a collection of independent contributed datasets (Table 1), all of which have been standardized to a common structure and units. Each dataset is given a reference name (internal to COSORE) that links its constituent tables, and provides a point of reference in reports. Each constituent dataset normally has a series of separate data tables:

- *description* (Table 2) describes site and dataset characteristics;
- *contributors* (Table 3) lists individuals who contributed to the measurement, analysis, curation and/or submission of the dataset;
- *ports* (Table 4) gives the different ports (generally equivalent to separate measurement chambers) in use, and what each is measuring: flux, species and treatment, as well as characteristics of the measurement collar;
- *data* (Table 5), the central table of the dataset, records flux observations;
- *ancillary* (Table S1) summarizes site‐level ancillary measurements;
- *columns* (Table S2) maps raw data columns to standard COSORE columns, providing a record for reproducibility; and
- *diagnostics* (Table S3) provides automatically generated statistics on the data import process: errors, columns and rows dropped, etc.

**TABLE 1:** Summary of COSORE v. 0.7.0 datasets with deposited data by International Geosphere‐Biosphere Programme land cover classification (Loveland et al., 2000) as provided by data contributors. Columns include number of datasets, total number of records (flux observations) and dates of first and last records

IGBP class	Datasets	Records	First record	Last record
Closed shrubland (CSH)	1	1,115	2013‐04‐01	2013‐05‐11
Cropland (CRO)	3	91,201	2016‐07‐17	2020‐02‐06
Deciduous broadleaf forest (DBF)	21	988,547	2003‐04‐20	2019‐12‐20
Deciduous broadleaf plantation (DBP)	1	11,337	2014‐03‐25	2014‐08‐31
Deciduous needleleaf forest (DNF)	2	153,495	2012‐09‐30	2018‐01‐01
Desert woodland (DWO)	1	11,581	2004‐01‐17	2004‐05‐07
Evergreen broadleaf forest (EBF)	11	1,477,747	2001‐12‐20	2017‐12‐12
Evergreen needleleaf forest (ENF)	18	2,944,839	2004‐01‐01	2019‐11‐11
Evergreen needleleaf plantation (ENP)	1	89,662	2009‐01‐21	2015‐12‐02
Grassland (GRA)	8	542,457	2005‐07‐19	2019‐11‐27
Mixed forests (MFO)	3	112,149	2006‐01‐01	2008‐02‐08
Open shrubland (OSH)	5	871,477	2005‐07‐22	2018‐11‐08
Savannas (SAV)	1	531,352	2015‐05‐22	2020‐02‐29
Wetland (WET)	4	180,868	2009‐07‐01	2017‐04‐21
Woody savanna (WSA)	4	129,437	2003‐06‐01	2020‐02‐12
(Total)	89	8,135,010	2001‐12‐20	2020‐02‐29

**TABLE 2:** Individual datasets in COSORE have a number of sub‐tables. The first of these is the *description* table, the fields of which are summarized below. Columns include field name, description, class (i.e. type of data), units and whether or not the field is required (required fields are marked by an asterisk)

Field name	Description	Class	Units	Req.
CSR_DATASET	Dataset name	character		*
CSR_SITE_NAME	Site name	character		*
CSR_LONGITUDE	Decimal longitude of site (positive = north)	numeric	degrees	*
CSR_LATITUDE	Decimal latitude of site (positive = east)	numeric	degrees	*
CSR_ELEVATION	Elevation of site	numeric	m	*
CSR_TIMEZONE	Site timezone code, from https://en.wikipedia.org/wiki/List_of_tz_database_time_zones	character		*
CSR_IGBP	Site IGBP class, from http://www.eomf.ou.edu/static/IGBP.pdf	character		*
CSR_NETWORK	Site network name	character		
CSR_SITE_ID	Site ID in network	character		
CSR_INSTRUMENT	Measurement instrument (i.e. model)	character		*
CSR_MSMT_LENGTH	Length of a single measurement	numeric	s	*
CSR_FILE_FORMAT	Raw data file format	character		*
CSR_TIMESTAMP_FORMAT	Raw data timestamp format, in R’s strptime() format	character		*
CSR_TIMESTAMP_TZ	Instrument timestamp timezone; usually but not always the same as CSR_TIMEZONE. From https://en.wikipedia.org/wiki/List_of_tz_database_time_zones	character		*
CSR_PRIMARY_PUB	Primary publication (DOI or URL)	character		
CSR_OTHER_PUBS	Other publications (DOI or URL)	character		
CSR_DATA_URL	Data link (DOI or URL)	character		
CSR_ACKNOWLEDGMENT	Acknowledgment text	character		
CSR_NOTES	Miscellaneous notes	character		
CSR_EMBARGO	Embargo flag. If this field is present, data will not be released	character		

**TABLE 3:** Summary of COSORE’s *contributors* table, which provides information on the researchers (at least one; there may be arbitrarily many listed) who measured and contributed each dataset. Columns include field name, description, class (i.e. type of data), units and whether or not the field is required (required fields are marked by an asterisk)

Field name	Description	Class	Units	Req.
CSR_FIRST_NAME	First (personal) name	character		
CSR_FAMILY_NAME	Family name	character		
CSR_EMAIL	Email address	character		
CSR_ORCID	ORCID ID; see https://orcid.org	character		
CSR_ROLE	CReDiT role; see https://www.casrai.org/credit.html	character		

**TABLE 4:** Summary of COSORE’s *ports* table, which provides information on the various multiplexed chambers that are frequently connected to a single measurement analyser. Columns include field name, description, class (i.e. type of data), units and whether or not the field is required

Field name	Description	Class	Units	Req.
CSR_PORT	Port (chamber) number; ‘0’ means all ports	integer		*
CSR_MSMT_VAR	Flux should be interpreted as: ‘Rs’ (soil respiration, whether CO_2_ or CH_4_), ‘Rh’ (heterotrophic respiration only), ‘Reco’ (ecosystem respiration), or ‘NEE’ (net ecosystem exchange)	character		*
CSR_TREATMENT	Chamber treatment; default is ‘None’	character		*
CSR_AREA	Area of measurement chamber	numeric	cm^2^	
CSR_VOLUME	Volume of measurement chamber	numeric	cm^3^	
CSR_DEPTH	Depth of collar insertion	numeric	cm	
CSR_OPAQUE	Opaque chamber?	logical		*
CSR_PLANTS_REMOVED	Plants removed from chamber?	logical		*
CSR_FAN	Mixing fan in chamber?	logical		
CSR_SPECIES	Comma‐separated species list	character		
CSR_SENSOR_DEPTHS	Comma‐separated list of sensor depths	character	cm	
CSR_LONGITUDE	Decimal longitude of measurement chamber, positive = north	numeric	degrees	
CSR_LATITUDE	Decimal latitude of measurement chamber, positive = east	numeric	degrees	
CSR_ELEVATION	Elevation of measurement chamber	numeric	m	

**TABLE 5:** Summary of COSORE’s *data* table, which holds the actual flux observations and accompanying time‐stamped data. Columns include field name, description, class (i.e. type of data), units and whether or not the field is required (required fields are marked by an asterisk); although not indicated, at least one flux observation (CSR_FLUX_CO_2_ or CSR_FLUX_CH_4_) is required in every database row. Note that all data in this table are acquired at the point of GHG flux measurement; see Table S1 for site‐level data

Field name	Description	Class	Units	Req.
CSR_DRY_CO_2_	Chamber CO_2_ concentration during flux measurement	numeric	ppmv	
CSR_DRY_CH_4_	Chamber CH_4_ concentration during flux measurement	numeric	ppbv	
CSR_CO_2__AMB	Ambient CO_2_ concentration at measurement chamber	numeric	ppmv	
CSR_CH_4__AMB	Ambient CH_4_ concentration at measurement chamber	numeric	ppbv	
CSR_COMMENTS	Comments	character		
CSR_CRVFIT_CO_2_	CO_2_ flux computation method (‘Lin’ or ‘Exp’ for linear and exponential)	character		
CSR_CRVFIT_CH_4_	CH_4_ flux computation method (‘Lin’ or “Exp” for linear and exponential)	character		
CSR_ERROR	Error raised by instrument or during import	logical		
CSR_FLUX_CO_2_	CO_2_ flux (positive = to atmosphere)	numeric	µmol CO_2_ m^−2^ s^−1^	
CSR_FLUX_CH_4_	CH4 flux (positive = to atmosphere)	numeric	nmol CH_4_ m^−2^ s^−1^	
CSR_FLUX_SE_CO_2_	Standard error of CO_2_ flux	numeric	µmol CO_2_ m^−2^ s^−1^	
CSR_FLUX_SE_CH_4_	Standard error of CH_4_ flux	numeric	nmol CH_4_ m^−2^ s^−1^	
CSR_LABEL	Port/chamber label	character		
CSR_PAR	Photosynthetically active radiation inside measurement chamber	numeric	µmol photons m^−2^ s^−1^	
CSR_PAR_AMB	Photosynthetically active radiation outside measurement chamber	numeric	µmol photons m^−2^ s^−1^	
CSR_PORT	Port/chamber number	integer		*
CSR_PRECIP	Precipitation at measurement chamber	numeric	mm	
CSR_R2_CO_2_	CO_2_ flux computation R2	numeric	fraction	
CSR_R2_CH_4_	CH_4_ flux computation R2	numeric	fraction	
CSR_RECORD	Record number within file	integer		
CSR_RH	Chamber relative humidity	numeric	%	
CSR_SMx	Volumetric soil moisture at × cm (other CSR_SMx fields follow same format)	numeric	m^3^/m^3^	
CSR_SOIL_O_2_	Soil oxygen level at measurement chamber	numeric	%	
CSR_Tx	Soil temperature at × cm (other CSR_Tx fields follow same format)	numeric	°C	
CSR_TAIR_AMB	Ambient air temperature at measurement chamber	numeric	°C	
CSR_TAIR	Chamber air temperature	numeric	°C	
CSR_TWATER	Groundwater temperature at measurement chamber	numeric	°C	
CSR_TIMESTAMP_BEGIN	Timestamp of beginning of flux observation, written YYYY‐MM‐DD HH:MM:SS	POSIXct		*
CSR_TIMESTAMP_END	Timestamp of end of flux observation, written YYYY‐MM‐DD HH:MM:SS	POSIXct		*
CSR_VPD	Vapour pressure deficit at measurement chamber	numeric	Pa	
CSR_WTD	Water table depth at measurement chamber, positive numbers are depth	numeric	cm	

The common key linking these dataset tables is the CSR_DATASET field, which records the unique name assigned to the dataset. In addition, a CSR_PORT key field links the *ports* and *data* tables. These links make it straightforward to extract datasets that have measured particular fluxes in certain ecosystem types, or isolate only non‐treatment (control) chamber fluxes, for example.

### 2.2 Versioning and archiving

COSORE uses semantic versioning (https://semver.org/), meaning that its version numbers generally follow an ‘*x*.*y*.*z*’ format, where *x* is the major version number (changing only when there are major changes to the database or package structure and/or function, in a manner that may break existing scripts using the data); *y* is the minor version number (typically changing with significant data updates); and *z* the patch number (bug fixes, documentation upgrades or other changes that are completely backwards compatible). Following each official (major) release, a DOI will be issued and the data permanently archived by Zenodo (https://zenodo.org/). All changes to the data or codebase are immediately available through the GitHub repository, but only official releases will be issued a DOI; we anticipate this happening on an approximately annual basis.

### 2.3 Data license and citation

The database license is CC‐BY‐4 (https://creativecommons.org/licenses/by/4.0/); see the ‘LICENSE’ file in the repository. This is identical to that used by e.g. FLUXNET Tier 1 and ICOS RI. In general, this license provides that users may copy and redistribute the database and R package code in any medium or format, adapting and building upon them for any scientific or commercial purpose, as long as appropriate credit is given. We request that users cite this article and strongly encourage them to (a) cite all constituent dataset primary publications, and (b) involve data contributors as co‐authors whenever possible, as is commonly done for other global databases such as FLUXNET (Baldocchi et al., 2001; Knox et al., 2019). In addition, users should also reference the specific version of the dataset they used (e.g. v0.6.0), access date and ideally the specific Git commit number. This supports reproducibility of any analyses.

## 3 DATA ACCESS AND USE

Major COSORE data releases are available via Zenodo (as noted above), as well as the GitHub ‘Releases’ page at https://github.com/bpbond/cosore/releases; we anticipate that institutional repositories such as ESS‐DIVE (Environmental Systems Science Data Infrastructure for a Virtual Ecosystem, https://ess‐dive.lbl.gov/) may host releases at some point in the future. Downloads via this page are flat‐file CSV (comma‐separated values), and readable by any modern computing system. Missing values are encoded by a blank (i.e. two successive commas in the CSV format). A release download is fully self‐contained, with full data, metadata and documentation; a file manifest; a copy of the data license; an introductory vignette; a summary report on the entire database; and an explanatory README with links to this publication.

An alternative way to access COSORE data, including minor updates between major releases, is to install and use the *cosore* R (R Core Team, 2019) package. This provides a robust framework, including dedicated access functions, dataset and database report generation and quality assurance and checking (see below). Because the flux data are currently included in the repository itself, the latter is quite large (compared to most Git repositories), ~215.4 MB. (Note that the data are stored in R’s compressed RDS file format; when loaded into memory, the entire database is significantly larger, ~565 MB.) It thus cannot easily be hosted on CRAN (the Comprehensive R Archive Network), the canonical source for R packages. Installing directly from GitHub is however straightforward using the *devtools* or *remotes* packages:





devtools::install_github("bpbond/cosore")

library(cosore)





**FIGURE 1:**
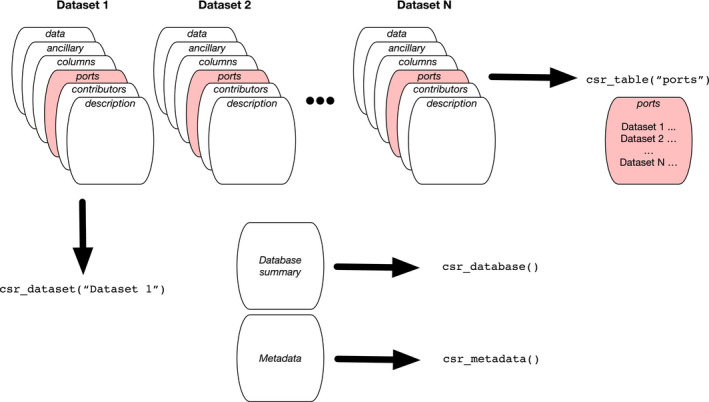
Summary of COSORE structure (multiple datasets, each with six tables; Tables 2, 3, 4, 5) and primary accessor R functions, as described in the text (see Section 2.1 in text). For example, R users can join specific tables across all datasets using the *csr_table()* function, and can access individual datasets with *csr_dataset()*. Non‐R users access flat‐file versions of the same data, with essentially the same structure as the R internal structure shown here

Four primary user‐facing functions (cf. Figure 1) are available:

- *csr_database()* summarizes the entire database in a single convenient data frame, with one row per dataset, and is intended as a high‐level overview. It returns a selection of variables summarized in Tables 2, 3, 4, 5 and Tables S1–S3, including dataset name, longitude, latitude, elevation, IGBP code, number of records, dates and variables measured;
- *csr_dataset()* returns a single dataset: an R list structure, each element of which is a table (*description*, *contributors*, etc., as described above);
- *csr_table()* collects, into a single data frame, one of the tables of the database, for any or all datasets;
- *csr_metadata()* provides metadata information about all fields in all tables.

Two additional reporting functions may also be useful to users:

- *csr_report_database()* generates an HTML report on the entire database: number of datasets, locations, number of observations, distribution of flux values, etc.;
- *csr_report_dataset()* generates an HTML report on a single dataset, including tabular and graphical summaries of location, flux data and diagnostics.

Finally, a number of functions are targeted at developers, and include functionality to ingest contributed data, standardize data and prepare a new release. See the package documentation for more details.

### 3.1 Documentation

The primary documentation for the COSORE database is this manuscript. Both the flat‐file releases and *cosore* R package include extensive documentation, including an in‐depth vignette included both in the package and online (https://rpubs.com/bpbond/502069). The R package includes documentation available via R’s standard help system.

### 3.2 Data quality and testing

When contributed data are imported into COSORE, the package code performs a number of quality assurance checks. These include:

- Timestamp errors, for example illegal dates and times for the specified time zone;
- Bad email addresses or ORCID identifiers;
- Records with no flux value;
- Records for which the analyzer recorded an error condition.

Any errors flagged or records removed during this process are summarized in the diagnostics table that is part of each dataset (Table S3 below). Across all contributed datasets, a median of 7.9% of raw observations were removed for one of these reasons. Note however that no checking on the flux values themselves is performed (e.g. for outliers, improbable values); currently this is the responsibility of the user.

The *cosore* R package also has a wide variety of unit tests (Zhao, 2003) that test code functionality via assertions about function behaviour and by verifying behaviour of those functions when importing test datasets (of different formats and with a variety of errors, for example). In total these tests cover 97.8% of the codebase.

## 4 CURRENT DATA AND COMMUNITY CONTRIBUTIONS

**FIGURE 2:**
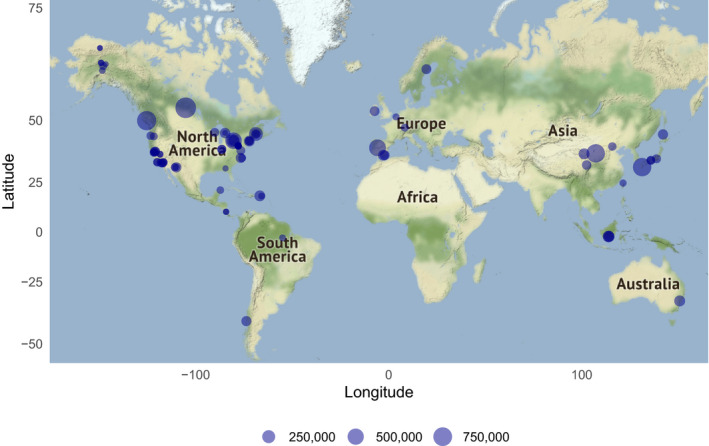
Geographic distribution of COSORE datasets (*N* = 89), with point sizes corresponding to the number of records in each dataset. Map tiles show USGS land cover and national elevation data and are by Stamen Design, under CC BY 3.0; data by OpenStreetMap, under ODbL; figure rendered using R’s *ggmap* (Kahle & Wickham, 2013)

**FIGURE 3:**
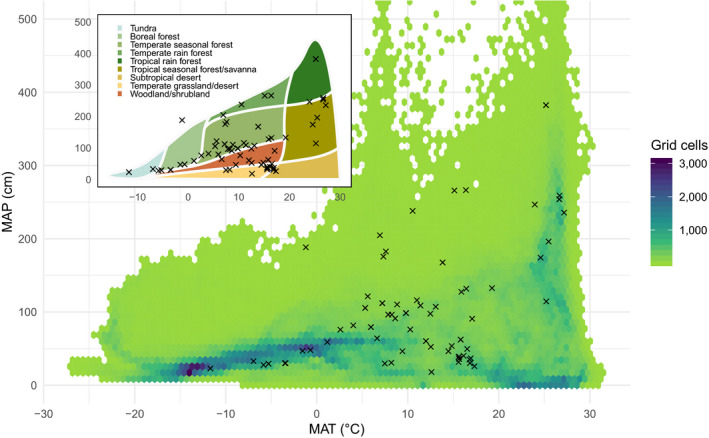
Distribution of COSORE datasets (black markers) in global climate space (WorldClim 2, Fick & Hijmans, 2017) of mean annual temperature (MAT) versus mean annual precipitation (MAP). Background colours indicate the number of half‐degree grid cells with each particular MAT–MAP combination. Inset plot shows the same points in Whittaker biome space (Ricklefs, 2008)

**FIGURE 4:**
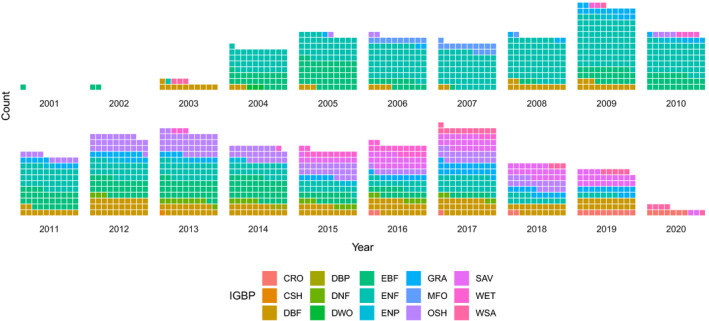
Flux observations, by IGBP (defined in Table 1), over time. Each square represents 5,000 observations, with categories of <5,000 observations rounded up so that they occupy a single square

**FIGURE 5:**
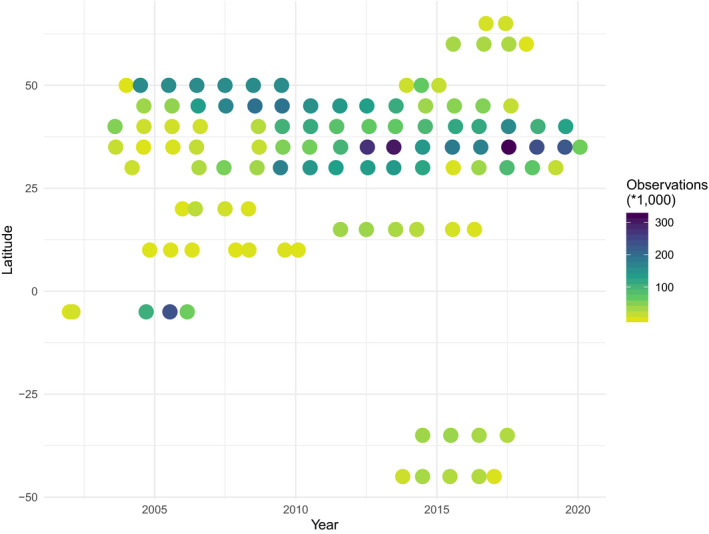
Temporal density of COSORE datasets, by latitude of the observational site

**FIGURE 6:**
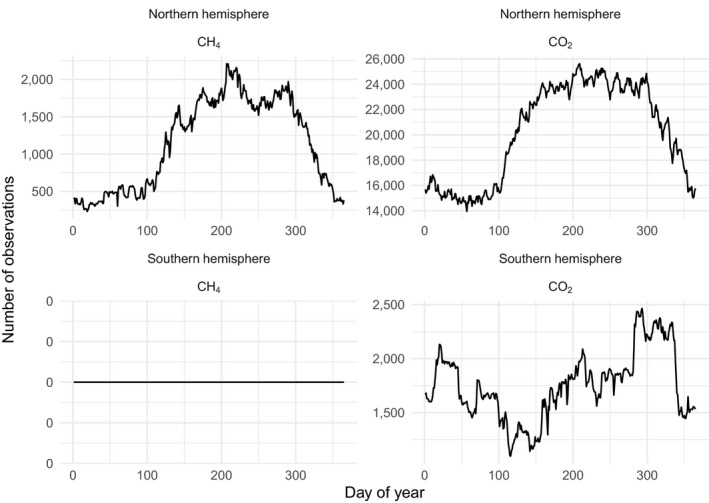
Number of observations by day of year, for northern and southern hemisphere and by gas (CO_2_ or CH_4_), in the current COSORE datasets; the database currently has no CH_4_ data from the Southern hemisphere (bottom left)

**FIGURE 7:**
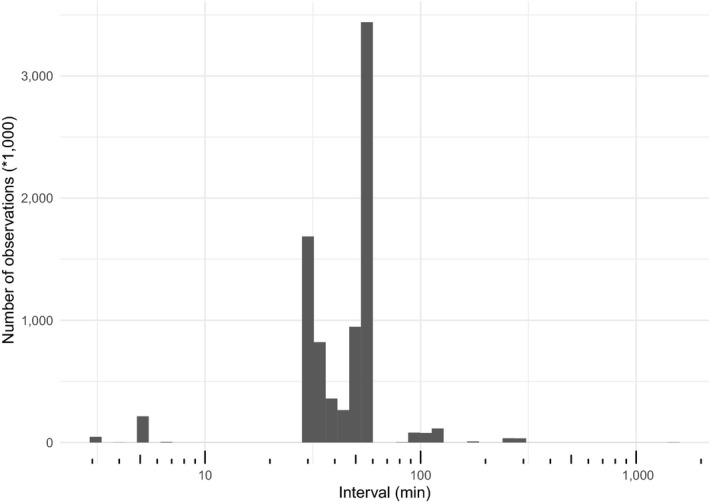
Temporal resolution (time interval between successive measurements, minutes; note logarithmic scale of *x*‐axis) of COSORE data

The database currently has 89 contributed datasets with a total of 8.14 million flux observations across 20 years and five continents (Table 1; Figure 2), widely distributed in climate and biome space, from Arctic to tropical ecosystems (Figure 3). In terms of data volume, the current database is dominated by CO_2_ fluxes in evergreen and deciduous forests (Table 1; Figure 4) from the mid‐northern latitudes (Figures 2 and 5). These data are unequally distributed around the year, with many more data available during the Northern Hemisphere growing season (Figure 6). There is an order of magnitude more data in COSORE from the Northern than Southern Hemisphere, and currently no CH_4_ data at all from the Southern Hemisphere. The interval between measurements ranges from 3 to 1,440 min, with 25%–50%–75% quantile values of 30, 60 and 60 min respectively. A one hour interval between measurements is thus by far the most common choice (Figure 7). Currently 92% of the datasets, and 99.999% of the data, provide sub‐daily temporal resolution. Such resolution allows novel analyses of the ‘hot moments’ of CO_2_ and other GHG fluxes (e.g. Diefenderfer et al., 2018).

**FIGURE 8:**
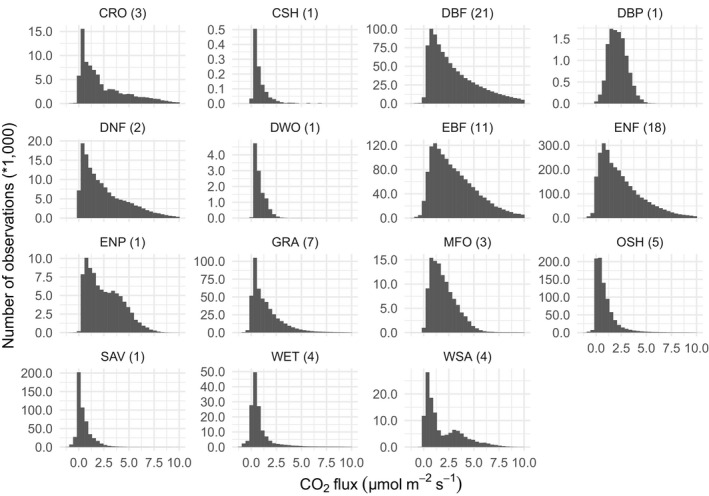
Distribution of CO_2_ fluxes in COSORE datasets, by IGBP classification (cf. Table 1). For visual clarity this figure excludes fluxes <−1 and >10 µmol m^−2^ s^−1^ (210,752 observations, 2.6% of the data). Number of datasets (sites) making up data is given in parentheses after IGBP abbreviations in each panel

**FIGURE 9:**
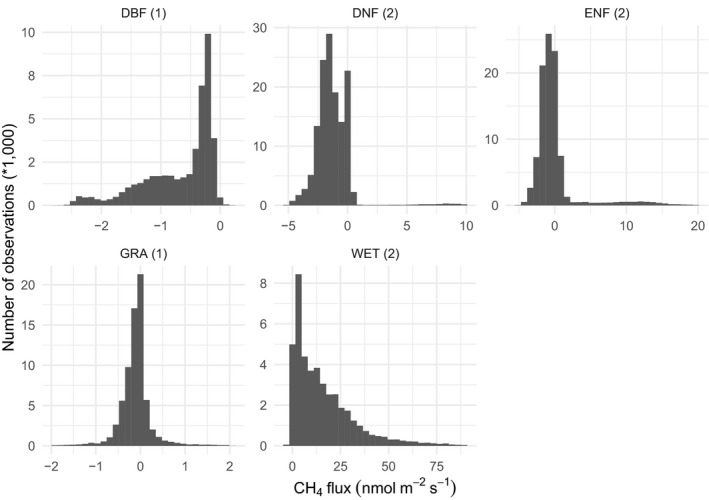
Distribution of CH_4_ fluxes in COSORE datasets, by IGBP classification (cf. Table 1). For visual clarity this figure excludes some extreme values (18,719 observations or 4.5% of the data). Number of datasets (sites) making up data is given in parentheses after IGBP abbreviations in each panel. Positive values are emissions to the atmosphere, and negative values uptake by the soil

Dataset CO_2_ fluxes (mostly soil respiration, but as noted above also some heterotrophic respiration and net ecosystem exchange) are generally log‐normally distributed in most IGBP classifications (Figure 8). The distribution of CH_4_ is more complex, with most data clustered around 0 nmol m^−2^ s^−1^ but featuring long distribution tails to many orders of magnitude larger fluxes for both net uptake and release (Figure 9), due to the complexity and variety of biochemical processes involved in methane production and oxidation (Riley et al., 2011).

The COSORE team welcomes data contributions of soil‐atmosphere GHG flux data. We prioritize continuously measured (i.e. from automated systems including non‐chamber approaches) soil respiration datasets, but the database structure also accommodates (discontinuous, i.e. manual) data, as well as measurements of methane, net ecosystem exchange and heterotrophic respiration fluxes. Contributors receive a QA/QC report for all submissions, including details on invalid data, removed data, etc., and can then request corrections or changes before the data are uploaded and go ‘live’; contributors may also request a temporary embargo on their data. There currently is no standardized data template that contributors must follow, but we anticipate this changing before version 1.0 (planned for late 2020). There is no minimum data coverage required, either in time or space, although we suggest datasets should at a minimum span a growing season.

It is important to note that COSORE itself is not (yet) a permanent data repository: it is an open community database, but not institutionally backed in the manner of Figshare (https://figshare.com), DataONE (https://www.dataone.org), ESS‐DIVE (https://ess‐dive.lbl.gov/) or ORNL‐DAAC (https://daac.ornl.gov). Its design reflects extensive consultation with many of these groups for seamless interoperability and perhaps future merging. Nonetheless, currently we recommend that contributors deposit data in such a repository first, and provide its Digital Object Identifier (DOI) in the COSORE dataset metadata.

We use the GitHub issue tracker (https://github.com/bpbond/cosore/issues) to track and categorize user improvement suggestions, problems or errors with the R package code or database data, requests for new variables or functionality and/or asking questions on any other aspect of COSORE. The COSORE team welcomes questions, contributions and suggestions (see the ‘CODE_OF_CONDUCT.md’ file in the repository).

## 5 CONCLUSIONS: STRENGTHS, LIMITATIONS AND FUTURE DIRECTIONS

COSORE is a ‘coalition of the willing’ (sensu Novick et al., 2018), and intended to be a community‐driven resource for analyses of soil‐atmosphere GHG exchange. Possible analyses and next steps include syntheses, model evaluation and methodological developments, e.g. in gap filling algorithms (Gomez‐Casanovas et al., 2013; Zhao et al., 2020). Soil‐atmosphere GHG flux measurements can be used at individual sites to check and constrain estimates of other carbon cycle fluxes (Miao et al., 2017; e.g. Phillips et al., 2017). Aggregated data across multiple ecosystems can be used to test proposed conceptual frameworks and model structures for expanding our understanding beyond first‐order temperature driven responses, and improving representation of *R*_S_ and other GHG fluxes in global ecosystem models (Abramoff et al., 2017; Mitra et al., 2019; Subke & Bahn, 2010). Finally, open data and open‐source harmonization tools (with which to compile disparate datasets) support scientific reproducibility, serve as an educational resource (Mouromtsev & d’Aquin, 2016) and reduce loss of data over time (Powers & Hampton, 2018).

A crucial attribute of COSORE is its relationship to preexisting databases and efforts. The older Global Soil Respiration Database (SRDB, Bond‐Lamberty & Thomson, 2010) focuses on seasonal to annual fluxes, with monthly‐ and daily‐resolution offshoots of the SRDB (Jian, Steele, Day, et al., 2018; Jian, Steele, Thomas, et al., 2018) following similar designs. Others, such as ForC (Anderson‐Teixeira et al., 2018), take a broader scope and also focus on annual fluxes. We hope that the large volume of standardized, high‐frequency GHG flux data in COSORE will enable novel global scale syntheses, modelling activities, new insights driven by machine learning (Albert et al., 2017; Vargas et al., 2018) and conceptual advances (e.g. Petrakis et al., 2017) that are currently impossible. Linking COSORE data with other high‐resolution, open databases such as FLUXNET (Baldocchi et al., 2001) and the ICOS RI Carbon Portal (https://www.icos‐cp.eu/data‐services) is also likely to yield new insights.

COSORE has a number of limitations, some peculiar to the effort and others intrinsic to the discipline and community. First, as with many observations in the ecological and Earth sciences, it is spatially non‐representative at the global scale (Xu & Shang, 2016), and currently dominated by datasets from North America and East Asia (Figure 2). There are no datasets from Africa (cf. Epule, 2015) and little South American data. The IGBP representation is skewed as well (Figure 4), although the database's climate space coverage is reasonable (Figures 3 and 6). This spatial patchiness—a function of many factors including economic development, infrastructure, scientific investment—imposes significant restrictions on our ability to draw global inferences and analyses from extant observational data.

A second category of limitations arises from COSORE’s particular design. The database is oriented towards lightweight and minimal requirements, aiming for breadth over depth. This has benefits and costs. Having low barriers to entry shifts the burden of contributing data away from data providers, and keeping the design lightweight (with limited controlled vocabularies, ancillary data, etc.) has kept the burden on COSORE’s designers and maintainers manageable; we are acutely aware that every additional field or piece of information imposes a cost, both immediately (for implementation) and in perpetuity (for maintenance). This was the rationale behind focusing initially on previously uncollated *continuous* measurements: to maximize scientific impact in terms of labour involved. In fact, nothing in COSORE’s design itself precludes incorporation of spatially distributed, survey‐style measurements. COSORE also remains relatively immature, with e.g. no ‘level 2’ data product incorporating external data (e.g. Fick & Hijmans, 2017). This imposes an additional cost—of time and effort—on database users to locate and integrate externally available data themselves.

Finally, analyses using COSORE will be limited by the nature of soil respiration and other soil‐atmosphere gas flux measurements, and the state of the disciplines’ networks and community. Automated measurements trade space for time: the systems are more expensive and require dedicated power, and do not perform well under certain conditions, limiting their spatial and temporal coverage at many scales (Barba et al., 2018). There remains no institutionally backed network akin to AmeriFlux or ICOS, and while there have been efforts to integrate chamber flux data into these networks’ data products, this has inevitable consequences for continuity and consistency. There is also no standardization of measurement depths for ancillary measurements (e.g. soil temperature and moisture) in the manner of a top‐down network such as NEON (Schimel et al., 2007) or ICOS RI (Op de Beeck et al., 2018).

### 5.1 Future directions

As noted above, every expansion or addition to a database imposes both immediate development costs and unending maintenance costs. Nonetheless, there are some areas into which COSORE could be expanded. Many automated systems record isotopes and H_2_O in addition to CO_2_ and/or CH_4_, and these data could be incorporated at relatively low cost; N_2_O and NH_3_ are other frequently measured GHGs. As noted above, downstream users would also benefit in the future from COSORE data premerged with global climate, ecological, field inventory or remote sensing data products. This feature is provided by the International Soil Radiocarbon Database (Lawrence et al., 2020), for example.

Currently, the COSORE team accepts flux data in any tabular format and performs unit conversion, restructuring and/or reformatting, etc., as needed. This was useful in the database's initial stages, as minimizing the work for contributors meant increased submissions. We intend however to shift this responsibility to data contributors before version 1.0, providing a template form that contributors must follow. This will allow for semiautomated data ingestion and follows the practices of many other earth sciences databases. Unusual or outlier measurements could also be automatically flagged for downstream users. More ambitiously, we have put substantial design work into ensuring interoperability so that COSORE data should flow relatively seamlessly into (or from) ESS‐DIVE, Ameriflux and ICOS RI. A long‐term vision is that COSORE data could, for example, automatically be made available in the larger community database. It is crucial, we believe, that COSORE contributors have assurances that their data contributions are traceable across versions and that it is not necessary to prepare and submit their data to multiple repositories. Finally, currently all data are included in the COSORE R package download. While convenient for users, this model will likely break down when the database doubles or triples in data volume. At that point, the data will need to be hosted elsewhere and downloaded only on demand.

## Supporting information

Tables_S1‐S3

## Data Availability

The data and code that support the findings of this study are openly available on GitHub at https://github.com/bpbond/cosore.
