# Supplementary material for: COSORE: A community database for continuous soil respiration and other soil‐atmosphere greenhouse gas flux data
Source: Glob Chang Biol. 2020 Oct 7;26(12):7268–83. doi: 10.1111/gcb.15353 (PMC7756728; doi:10.1111/gcb.15353)
Supplement: Supplementary file 1 — Tables_S1‐S3 [file GCB-26-7268-s001.docx]

**Supplementary Table S1.** Summary of COSORE’s *ancillary* table, which includes optional information, typically ecosystem-level soil information, carbon fluxes, and climate normals. Columns include field name, description, class (i.e. type of data), units, and whether or not the field is required. Vegetation fields (CSR_ANPP, etc.) refer to all species combined. The date/time of the observation is required, either the timestamp variables (CSR_TIMESTAMP_BEGIN and CSR_TIMESTAMP_END) or the date variable (CSR_DATE). Note that all data in this table apply to the entire field site; see **Table 5** for data co-located with the flux measurements.

| **Field name** | **Description** | **Class** | **Units** | **Req.** |
| --- | --- | --- | --- | --- |
| CSR_TIMESTAMP_BEGIN | Timestamp of beginning of ancillary observation (YYYY-MM-DD HH:MM:SS) | POSIXct | ISO 8601 date-time |  |
| CSR_TIMESTAMP_END | Timestamp of end of ancillary observation (YYYY-MM-DD HH:MM:SS) | POSIXct | ISO 8601 date-time |  |
| CSR_DATE | Date (YYYY or YYYY-MM or YYYY-MM-DD) of which ancillary measurements are representative; this and the timestamps fields should not both be filled out | character |  |  |
| CSR_STATISTIC | Statistic being reported in this row, following AmeriFlux controlled vocabulary (Mean, Max, Standard Deviation, Measurement Uncertainty, Single observation, Expert estimate, etc.) | character |  |  |
| CSR_ABM | Aboveground biomass | numeric | gC/m2 |  |
| CSR_AGE | Time since most recent disturbance | numeric | years |  |
| CSR_ANPP | Total aboveground net primary production | numeric | gC/m2/yr |  |
| CSR_BA | Basal area | numeric | m2/ha |  |
| CSR_BD | Soil bulk density | numeric | g/cm3 |  |
| CSR_BNPP | Total belowground net primary production | numeric | gC/m2/yr |  |
| CSR_CLAY | Clay | numeric | % |  |
| CSR_CN | Soil carbon to nitrogen ratio | numeric |  |  |
| CSR_DEPTH | Depth of soil measurements | numeric | cm |  |
| CSR_ER | Ecosystem respiration | numeric | µmol/m2/s |  |
| CSR_GPP | Gross primary production | numeric | µmol/m2/s |  |
| CSR_LAI | Leaf area index | numeric | m2/m2 |  |
| CSR_LITTERFALL | Litterfall dry mass | numeric | g/m2/yr |  |
| CSR_MAT | Mean annual air temperature | numeric | °C |  |
| CSR_MAP | Mean annual precipitation | numeric | mm/yr |  |
| CSR_NEE | Net ecosystem exchange (negative = carbon sink) | numeric | µmol/m2/s |  |
| CSR_NEP | Net ecosystem production, typically biometric (positive = carbon sink) | numeric | gC/m2/yr |  |
| CSR_NH4 | Ammonium, dry soil | numeric | mg/kg soil |  |
| CSR_NO3 | Nitrate, dry soil | numeric | mg/kg soil |  |
| CSR_NPP | Total (above + below) net primary production | numeric | gC/m2/yr |  |
| CSR_PAR | Photosynthetically active radiation | numeric | µmol/m^2^/s |  |
| CSR_PH | Soil pH | numeric |  |  |
| CSR_PRECIP | Precipitation at site | numeric | mm |  |
| CSR_SAND | Sand | numeric | % |  |
| CSR_SILT | Silt | numeric | % |  |
| CSR_SOC | Soil organic carbon | numeric | gC/m^2^ |  |
| CSR_STC | Total soil carbon | numeric | % |  |
| CSR_STN | Total soil nitrogen | numeric | % |  |
| CSR_VPD | Vapor pressure deficit | numeric | Pa |  |
| CSR_WIND | Wind speed | numeric | m/s |  |
| CSR_WTD | Water table depth (positive numbers = depth) | numeric | cm |  |

**Supplementary Table S2.** Summary of COSORE’s *columns* table, which maps raw dataset columns to standardized COSORE columns. Columns include field name, description, class (i.e. type of data), units, and whether or not the field is required. We expect that this table will be dropped at some point in the future when COSORE requires structurally compliant data submissions (i.e. contributors will be required to format their data to match COSORE structure before submission).

| **Field name** | **Description** | **Class** | **Units** | **Req.** |
| --- | --- | --- | --- | --- |
| Database | Database column name (e.g. CSR_FLUX_CO2) | character |  | * |
| Dataset | Dataset columns name | character |  | * |
| Computation | Optional R computation to be performed changing units, etc. | character |  |  |
| Notes | Notes | character |  |  |

**Supplementary Table S3.** Summary of COSORE’s *diagnostics* table, which is populated automatically when parsing and importing non-COSORE data. Columns include field name, description, class (i.e. type of data), units, and whether or not the field is required.

| **Field name** | **Description** | **Class** | **Units** | **Req.** |
| --- | --- | --- | --- | --- |
| CSR_ASSUMED_MSMT_LENGTH | If no measurement length is provided this is set to assumed value | numeric | s |  |
| CSR_EXAMPLE_BAD_TIMESTAMPS | Example bad timestamps | character |  |  |
| CSR_GASES | Gases present in final dataset | character |  | * |
| CSR_RECORDS | Records in final dataset | integer |  | * |
| CSR_RECORDS_REMOVED_ERR | Number of observations removed for error code | integer |  | * |
| CSR_RECORDS_REMOVED_NA | Number of observations removed for missing flux | integer |  | * |
| CSR_RECORDS_REMOVED_TIMESTAMP | Number of observations removed for bad timestamp | integer |  | * |
| CSR_TIMESTAMP_BEGIN | Timestamp of first flux observation, written YYYY-MM-DD HH:MM:SS | POSIXct |  | * |
| CSR_TIMESTAMP_END | Timestamp of last flux observation, written YYYY-MM-DD HH:MM:SS | POSIXct |  | * |
